# Pre-Exercise Hydration Modulates the Sweat Rate and Executive Control during Moderate Exercise to 3% Dehydration under Thermoneutral Conditions

**DOI:** 10.5114/jhk/220097

**Published:** 2026-04-02

**Authors:** Karol Skotniczny, Michał Toborek, Artur Terbalyan, Adam Zając, Mateusz Gawełczyk, Szymon Siatkowski, Jakub Chycki

**Affiliations:** 1 Institute of Sport Sciences, The Jerzy Kukuczka Academy of Physical Education in Katowice, Katowice, Poland.; 2 Department of Biochemistry and Molecular Biology, University of Miami Miller School of Medicine, Miami, Florida, United States of America.

**Keywords:** executive function, hydration status, thermoregulation, core temperature, exercise-induced dehydration

## Abstract

Dehydration impairs endurance and may compromise cognition, yet the impact of pre-exercise hydration status on thermoregulation and cognitive function during continuous exercise is not well defined. Thirty physically active men were randomized to be tested either well-hydrated (HYD; n = 16) or insufficiently hydrated (HYP; n = 14), classified by fasting urine specific gravity (USG; HYD < 1.018; HYP > 1.018) verified over three weeks. On the experimental day, participants cycled at 50% maximal power output (Wmax) under thermoneutral conditions (22°C, 45% RH) until 3% body-mass loss or for 120 min. Core temperature (Tc) was recorded continuously, the sweat rate (SR) was derived from nude body-mass change, and executive function (Stroop Interference) along with visuospatial working memory (Corsi Block-Tapping) were assessed pre- and post-exercise. Participants in the HYD group produced a higher SR (ΔSRmean +0.30 L·h⁻^1^, 95% CI 0.18–0.43; ΔSRpeak +0.34 L·h⁻^1^, 95% CI 0.04–0.63) and were more likely to reach 3% mass loss (10/16 vs. 1/14; p = 0.002). Despite greater sudomotor output, early Tc burden (0–90 min) did not differ between groups. Hydration status selectively affected executive control: Stroop naming interference showed a time × status interaction (F(1,27) = 4.57, p = 0.042), driven by slowing in HYP participants (≈+63 ms, p = 0.029), whereas Corsi indices were unchanged. These findings support targeting euhydration before prolonged exercise (e.g., morning USG <1.018), as inadequate baseline hydration may impair inhibitory control even when early Tc responses are comparable. Accordingly, for training or competition requiring rapid decision-making and attentional control, pre-exercise hydration strategies (planned fluid intake and/or USG-based monitoring) may be warranted to mitigate decrements in executive function.

## Introduction

The literature consistently demonstrates that dehydration detrimentally affects athletic performance, whereas sufficient hydration is a requisite for optimizing physiological processes underpinning sports performance. Even modest body-water deficits can elevate the heart rate and core temperature (Tc), increase perceived effort, and limit endurance performance. Consensus statements and quantitative reviews converge on a practical threshold of ~2% body-mass loss as the point at which impairments are consistently observed (Sawka et al., 2007). Accordingly, authoritative guidance emphasizes beginning exercise euhydrated and matching fluid intake to individual sweat losses. Beyond total volume, the mineral composition and alkalinity of ingested fluids can modulate hydration and acid-base status, with chronic intake of mineral-based alkaline water improving urine-based hydration indices, post-exercise lactate clearance, and repeated Wingate performance in trained athletes (Chiron et al., 2024; Chycki et al., 2017, 2018).

Epidemiological evidence suggests that hypo hydration is prevalent across various athletic contexts, both before and during training and competition (Magee et al., 2017). The National Athletic Trainers’ Association reports that a substantial proportion of athletes at the professional, collegiate, secondary-school, and youth levels commence exercise in a hypo hydrated state, and that ad libitum fluid intake during activity typically offsets only approximately two-thirds of sweat losses (McDermott et al., 2017). In collegiate sport, a cross-sectional study of NCAA Division I athletes reported 13% of them being significantly hypo hydrated (urine specific gravity [USG] ≥ 1.030) and 53% hypo hydrated (USG 1.020–1.029) before practice. In soccer, a quantitative systematic review pooling 24 studies found pre-exercise hypo hydration prevalence of 63.3% (by USG), 37.4% (urine osmolality), and 58.8% (urine color), with higher rates in men, professionals, and before training versus before matches (Volpe et al., 2009). Collectively, these findings underscore the need for proactive, sport- and athlete-specific hydration strategies in both daily training and competition.

During moderate-intensity exercise, metabolic heat production increases core temperature, making evaporative heat loss via sweating the primary avenue for maintaining thermal balance. Human thermoregulation is governed by a closed-loop control system that integrates afferent thermal signals with efferent heat-dissipating responses (Baker, 2019; Cramer et al., 2022; Powers and Howley, 2018). Central thermoreceptors within the brain and the spinal cord, together with cutaneous thermoreceptors, continuously sense core and skin temperatures. These inputs are integrated in the preoptic-anterior hypothalamus (POAH), which coordinates autonomic output in response to deviations from the defended core temperature (Xu et al., 2023). When Tc exceeds the resting set point (~37°C), the POAH increases sympathetic cholinergic sudomotor drive to eccrine glands and engages active cutaneous vasodilation, thereby elevating sweat secretion and the skin blood flow. Arteriolar dilation facilitates convective transfer of heat from the core to the body surface, while evaporation of sweat from the skin provides the principal avenue for heat loss (Kenney et al., 2021). As exercise continues, the skin blood flow and sweating progressively increase in proportion to the rising Tc (Gleeson, 1998). The sweat rate (evaporation rate) tends to increase linearly with further elevations in Tc above the threshold. Typical sweat rates during moderate exercise may be on the order of ~0.5–1.0 L/h, but vary widely with condition, as well-trained athletes or those subjected to intense exercise in hot environments can significantly exceed this value. Sweating rates of 2–3 L/h have been recorded in well-trained runners and soccer players under intense heat stress (Armstrong et al., 1986).

Hydration critically affects thermoregulation. If an athlete becomes dehydrated, two interrelated mechanisms impair heat dissipation: (i) contraction of plasma volume and (ii) increases in plasma osmolality. The reduction in central blood volume limits the fraction of cardiac output available for thermoregulatory skin blood flow, and hypo hydration elicits reflex cutaneous vasoconstriction, resulting in diminished perfusion of eccrine sweat glands and reduced sweat secretion and evaporative heat loss (Sawka and Pandolf, 1990). The dynamic interplay among rises in Tc, the magnitude of sweat loss, and pre-exercise hydration status are critical determinants of thermoregulatory efficacy and, by extension, sustained exercise performance.

Moreover, hypo hydration appears to redistribute the blood flow toward the periphery while attenuating cerebral perfusion, a combination associated with decrements in cognitive performance (Lieberman, 2007; Skalski et al., 2024, 2025a, 2025b). Additionally, dehydration may slow synaptic transmission within the prefrontal cortex, thereby reducing information-processing speed (Secher and Ritz, 2012). Converging evidence indicates that hypo hydration impairs higher-order cognition, with the most consistent effects observed for executive functions, including inhibitory control and working memory. Even mild dehydration (e.g., after 24 h fluid restriction) has been associated with slower responses on Stroop tasks (Watanabe et al., 2024; Skalski et al., 2025c, 2025d), whereas findings for visuospatial working memory assessed by the Corsi Block-Tapping test are more controversial: acute water ingestion often yielded small performance benefits, while exercise-induced hypo hydration in the ~2–5% body-mass range frequently produced negligible immediate decrements (Deshayes et al., 2022). Importantly, the cognitive response to exercise is not uniformly negative, as acute exercise itself may transiently enhance executive performance; for example, a single 15-min bout of high-intensity interval training improved Stroop-task response times and increased 2-back working-memory accuracy in healthy young adults compared with moderate-intensity interval exercise, moderate-intensity continuous exercise, and rest (Yue et al., 2025; Prończuk et al., 2024, 2025).

Meta-analysis likewise suggests that decrements are more likely when fluid loss exceeds ~2% body mass, though substantial heterogeneity highlights the importance of task choice, a dehydration method, thermal load, and individual differences (Wittbrodt and Millard-Stafford, 2018). Complementary evidence suggests that acid-base manipulation can affect post-exercise cognition: in elite combat-sport athletes, 21 days of sodium bicarbonate supplementation enhanced lactate efflux and working-memory speed during repeated Wingate testing, indicating that a peripheral metabolic state could modulate executive functioning (Chycki et al., 2021).

Despite robust evidence on exercise-induced dehydration and its consequences for thermoregulation and cognition, the influence of pre-exercise hydration status has been insufficiently studied. Therefore, the objective of this study was to examine the relationship among baseline hydration status, changes in Tc and sweat loss during moderate-intensity exercise performed to a target dehydration level of 3% of body mass. Physiological data were used to determine the dynamic interplay of exercise thermoregulatory response and baseline hydration status. Moreover, an additional objective was to verify the association between baseline hydration status and cognitive performance in the domains of executive functions and working memory under exercise-induced dehydration.

We hypothesized that hydration status would alter the temporal dynamics of Tc during constant-load exercise and that Tc trajectories would correlate with sweat-loss kinetics; specifically, larger increases in Tc would be associated with higher rates of sweat loss at moderate intensity. Additionally, we hypothesized that individuals with poorer baseline hydration status would exhibit lower cognitive performance and a greater decrement following further exercise-induced fluid loss.

## Methods

### Study Design

Consistently with the assumption that the vast majority of physically active individuals are at risk of chronic dehydration, participants were randomly (using the random.org List Randomizer) assigned to one of the two experimental groups: a well-hydrated (HYD) group or an insufficiently hydrated (HYP) group. Participants in the HYD (n = 16) group received specific guidance on hydration and post-exercise fluid replacement, whereas the HYP (n = 14) group was instructed to maintain their habitual fluid-intake practices. Across three weekly assessment points, hydration status was verified by fasting urine specific gravity (USG), confirming optimal hydration among HYD participants (mean USG < 1.018) and indicating a chronic dehydration risk among HYP participants (mean USG > 1.018). We operationalized hydration status with fasting urine specific gravity (USG). Although USG = 1.020 is commonly cited as a practical screening threshold, we adopted 1.018 a priori to improve sensitivity for mild hypo hydration risk in active adults and to align with our repeated-measures classification. Participants received verbal and written explanations of the protocol, were informed that they could withdraw at any time, and provided written consent to participate. The study had prior approval from the University Bioethics Committee for Scientific Research at the Jerzy Kukuczka Academy of Physical Education in Katowice, Katowice, Poland (approval code: 3-X/2023; approval date: 19 October 2023).

An experimental protocol was implemented to collect measures reflective of chronic dehydration and to profile signal sequences denoting uncompensated fluid loss under exercise conditions (dehydration of 3% body mass) in a physically active cohort to assess the relationship between changes in Tc and the rate of sweat loss considering different initial hydration states, as well as the impact on neurocognitive performance. Participants, at the intervention-qualification stage, underwent a physician-led screening to assess whether they met inclusion/exclusion criteria, completed maximal aerobic capacity testing (VO_2__max_), and were monitored for hydration status. The qualification and randomization phase lasted up to 4 weeks.

Aerobic capacity was assessed using a ramp protocol T20×1 (20 W·min⁻^1^). The standardized test involved a progressively increasing work rate with a resistance increment of 0.33 W·s⁻^1^. Tests were performed on an Excalibur Sport cycle ergometer (Lode), commenced at 40 W, and, at a supervised cadence of 70–80 rev·min⁻^1^, continued until VO_2__max_ was reached. Participants exercised to volitional exhaustion or until they could not maintain the target cadence. VO_2__max_ attainment was verified using the following criteria: (1) a VO_2_ plateau despite further workload increments (ΔVO_2_ < 150 mL·min⁻^1^), and (2) a respiratory exchange ratio (RER) > 1.10. At rest and throughout exercise, the heart rate (HR), minute ventilation (V̇E), oxygen uptake (V̇O_2_), and carbon dioxide output (V̇CO_2_) were recorded continuously using a MetaLyzer 3B-2R gas analyzer (Cortex). The determination of maximal power output (W_max_) constituted a critical step for prescribing relative intensity in the planned exercise intervention.

The intervention phase was conducted after the qualification stage and continued until the target sample (n = 30) was completed. Participants, up to three persons per day, visited the laboratory at 08:00 and underwent all procedures after a standardized meal. All participants consumed 3–5 ml/kg water upon waking. Before and after the dehydration protocol, participants underwent neurocognitive testing to evaluate executive functions and working memory. Exercise-induced dehydration was assessed by cycling on a stationary ergometer at 50% of the individual's W_max_ (determined during the qualification stage). The heart rate was monitored continuously (Polar T300), as well as Tc (Core, AG, Switzerland), and exercise continued until each participant reached a 3% body-mass reduction due to dehydration or for a maximum of 120 min, whichever occurred first. Measurements were catalogued in 15-min intervals. Sessions were conducted under thermoneutral conditions (22°C, RH 45%, air velocity <0.2 m·s⁻^1^; no solar load). Moreover, participants performed the exercise without an upper-body garment to avoid potential fabric-related effects on the rate of sweat loss. Exercise was paused every 15 min to quantify sweat loss via nude body mass measurement conducted in a private room. Before each weighing, participants were thoroughly towel-dried to remove residual sweat to minimize measurement error.

### Participants

Thirty physically active men (n = 30) participated in the study. Participants constituted a representative group, selected based on purposeful eligibility criteria (inclusion): (i) age 25–60 years; (ii) absence of obesity—body fat content (FM) <30%; (iii) absence of acute disease syndromes; (iv) provision of informed consent to participate. The exclusion criteria comprised: uncontrolled arterial hypertension, unstable coronary artery disease, cardiac arrhythmias, and the presence of a pacemaker, liver or kidney disease, and lack of consent to participate. With α = 0.05 and group sizes HYD: n = 16, HYP: n = 14, a sensitivity analysis (G*Power 3.1; Heinrich-Heine-Universität Düsseldorf, Düsseldorf, Germany; Faul et al., 2007) indicated 80% power only for medium-large adjusted effects; for typical pre-post correlations (r ≈ 0.50–0.70), the minimal detectable standardized difference was approximately d ≈ 0.76–0.92.

### Measures

The protocol contained diagnostic assessments before, during, and after exercise-induced dehydration. Evaluations included the assessment of morphostructural variables using dual-energy X-ray absorptiometry (DXA), evaluation of cognitive performance before and after the exercise intervention, hydration status, monitoring of changes in Tc during exercise, and determination of the sweat loss rate. The cataloging of multisource data was undertaken to evaluate the sequence of thermoregulatory changes, their impact on physiological and neurocognitive variables, and their interrelationships, while accounting for participants’ differing baseline hydration status.

### Body Composition Assessment (DXA)

Whole-body composition was evaluated by dual-energy X-ray absorptiometry (DXA) in a whole-body mode using a Lunar iDXA Advance scanner (GE Healthcare, USA) with K-edge-filtered dual energies (~39 and ~71 keV from a 100 kV generator). Two total-body scans were obtained with participants positioned supine per manufacturer procedures. The recorded output included body mass (BM, kg), lean body mass (LBM, kg), fat mass (FM, kg), fat mass percentage (FM, %), and bone mineral density (BMD, g/cm^2^), exported from the system software. The estimated effective dose per whole-body iDXA scan was ~0.96–1.92 µSv (thin/standard vs. thick mode), i.e., < 10 µSv overall (manufacturer data indicate a skin entrance dose for total-body scans <10 µGy). Whole-body DXA provides high short-term precision when standardized scanning and positioning procedures are used and in physically active adults, repositioning produces trivial error for whole-body outcomes, supporting test-retest reliability under controlled conditions (Nana et al., 2012).

### Cognitive Performance Assessment

All cognitive tests were administered at a dedicated station under standardized auditory/visual conditions using the Vienna Test System (Schuhfried, Austria), a psychometrically standardized computerized test battery. Executive function was assessed with the Stroop Test (SIT-S8) and working memory with the Corsi Block-Tapping Test. In the SIT-S8, the words “red”, “green”, “yellow”, and “blue” (5–7 mm × 10 mm) appeared on a black screen in one of four colors; 50% of trials were congruent and 50% incongruent. Starting 1,000 ms after the test onset, each stimulus remained until response; participants had 2,500 ms to press the matching color on a four-button panel using the index and middle fingers of both hands. After a 10-trial practice, two 60-trial diagnostic blocks were completed with no overall time limit. For both color-naming and word-reading parts, three interference indices were computed from the trial log. The Vienna Test System Stroop Interference Test (SIT) shows strong internal-consistency estimates (split-half reliability reported in the high range across derived indices) and evidence of construct validity based on known-group comparisons (Schuhfried GmbH, 2006).

Reaction-time interference (RTinterf) was defined as the within-participant difference in median reaction time between incongruent and congruent trials: RT_interf_ = median RT_incongruent_ − median RT_congruent_. To adjust this cost for accuracy, we computed an inverse-efficiency interference score (IES_interf_) as IES_interf_ = RTi_nter_ / (1 − *p_err_*) where *p*_err_ was the proportion of errors in the incongruent trials of that part. Given 30 incongruent trials per part, *p*_err_ = (number of errors) / 30. Error interference was the percentage of incorrect responses in the incongruent trials: Error interference = *p*_err_ × 100%. All indices were computed separately for naming and reading.

The Corsi Block-Tapping Test (Vienna Test System, Schuhfried GmbH) was administered to assess visuospatial short-term and working memory. In the computerized implementation, nine spatially separated squares were displayed in a fixed, irregular array on the screen. On each trial, a subset of squares was highlighted sequentially to form a spatial sequence. Participants reproduced the sequence by selecting the squares in order using the input device (mouse/touchscreen), following standardized on-screen instructions. After familiarization trials, testing commenced with a short sequence; sequence length increased by one after a correct reproduction. The test continued until the participant failed sequences of a given length according to the built-in stopping rule (e.g., two errors at the same length), at which point the condition terminated. The testing environment was standardized, and the response hand was not constrained. The subsequent statistical analyses used the Corsi Block-Tapping Test outcomes: block time (BT) (ms), scaled score (RSS), spatial working memory (SWM), best span, and the raw score as the primary continuous measures. Error-based metrics, i.e., the number of incorrect trials and order errors, were also analyzed (summarized as median [IQR]). The Vienna Test System CORSI Block-Tapping Test demonstrates good psychometric performance (reported reliability and evidence of discriminant validity in neurological samples), and de Paula et al. (2016) confirmed acceptable internal consistency for Corsi span outcomes.

### Hydration Assessment

At each study phase, hydration status was verified via urine specific gravity measured with an ORM 2UN refractometer (Kern & SOHN GmbH, Germany), which was calibrated with distilled water in accordance with the manufacturer’s instruction. Each participant underwent three assessments spaced at intervals of no less than one week. Initiation of the experimental intervention was permitted only if the hydration criteria specified by the randomized allocation, HYD or HYP, were fulfilled. Urine specific gravity is a commonly used urinary marker of hydration and shows strong correspondence with urine osmolality for classifying hydration status. Agreement studies also indicate that refractometer-based USG measurements demonstrate high concordance across devices, supporting measurement validity for screening purposes (Armstrong et al., 1994; Minton et al., 2015)

### Core Body Temperature (TC) and the Sweat Rate

Throughout the intervention, continuous Tc monitoring was performed using non-invasive CORE sensors (greenTEG AG, Switzerland). The CORE sensor estimates Tc non-invasively by combining measurements of cutaneous heat flux with skin temperature and environmental inputs. These signals are processed in real time by a proprietary algorithm that fuses bio thermal heat-transfer modeling with machine-learning calibration against reference methods. The device provides continuous, second-to-second estimates of Tc when affixed to the chest or the lateral torso according to manufacturer guidelines. Accuracy is condition-dependent and could be influenced by rapid thermal transitions, motion, and local skin perfusion (Daanen et al., 2023; Gagnon et al., 2010).

Sensors were mounted in accordance with the manufacturer’s instructions at sites enabling the most reliable readings (e.g., on the chest over the sternum or on the lateral surface of the torso). Data were monitored in real time via an application or a receiver device compatible with the sensors. Tc was recorded continuously (1 Hz) using CORE sensors. For inferential analyses, the continuous stream was down-sampled to 15-min epochs (pre, 15, 30, 45, 60, 75, 90 min) to align with scheduled weighing and minimize artefacts. Segments with motion artefacts or loss of telemetry were flagged and removed based on pre-specified thresholds (implausible Tc jumps >0.5°C·min⁻^1^, missingness >5 s).

Additionally, at standardized 15-min intervals, sweat loss was assessed using a floor scale. Measurements were obtained nude in a private room, and participants were instructed to thoroughly towel-dry their bodies to objectify the measurement. Measurements were recorded to monitor the kinetics of water loss and to evaluate the sequence of signals associated with changes in Tc and the sweat-loss rate.

### Nutrition and Hydration Guidelines

Following the completion of the qualification stage, the HYD group was provided with hydration guidance on a daily water intake of 2.5 L, in line with the current recommendations for adult men (EFSA Panel on Dietetic Products, 2010). With regard to the consumption of fluids in conjunction with the recommended daily intake, it was advised that a quantity of 5–7 ml/kg should be ingested four hours before physical exercise, with a further intake of 3–5 ml/kg two hours prior to exercise when the color of the urine indicated hypo hydration, as suggested by Sawka et al. (2007). It was also recommended to consume 200–300 ml of fluids every 15 minutes during exercise (Casa et al., 2000). Furthermore, each participant was to consume 120–150% of their body mass loss during the exercise, with the body mass difference measured before and after exercise.

A standardized breakfast, comprising 65% carbohydrates, 25% fats, and 15% proteins (approximately 4.5 kcal/kg body weight), was provided two hours prior to the beginning of the trial. Before testing, participants were requested to ingest a volume of fluids ranging from 3 to 5 ml/kg of their body weight, a requirement to be met two hours prior to the start of the trial. The consumption of any beverages was prohibited from the final measurement of body mass prior to the start of the test, through to the completion of the second round of cognitive performance tests and body composition assessment.

### Statistical Analysis

Descriptive statistics were reported according to their distribution: approximately normal variables as mean ± SD and/or mean with 95% confidence intervals (CI); skewed variables as median [IQR] (range). Post-90-min missingness was MNAR (Missing Not At Random) because stopping at 3% body-mass loss makes missingness depend on unobserved higher Tc/SR (informative dropout). To avoid MCAR/MAR (Missing Completely At Random/Missing At Random) bias, primary analyses were restricted to 0–90 min with no imputation. Derived variables were computed from 15-min samples (pre, 15, 30, 45, 60, 75, 90 min). The interval sweat rate (L·h⁻^1^) was calculated from body-mass change between consecutive 15-min body mass assessments divided by 0.25 h; summary indices were SR_mean_ (mean of available intervals) and SR_peak_ (maximum).

Assumptions were examined prior to inference: normality by Shapiro-Wilk and Q-Q plots of residuals, homogeneity of variances by the Levene’s test. Between-group comparisons used the Student’s *t*-test or the non-parametric Mann-Whitney U test for non-normal data. For *t*-tests, effect size was Cohen’s *d* with 95% CI, interpreted using conventional benchmarks (small ≈ 0.20, medium ≈ 0.50, large ≈ 0.80; Cohen, 1988). Time effects across repeated measures were assessed with the Friedman’s test; effect size was Kendall’s W (small ≈ 0.10, medium ≈ 0.30, large ≈ 0.50). Post-hoc paired comparisons used the Durbin-Conover procedure with Holm correction for multiplicity. Cognitive endpoints were analyzed via repeated measures ANCOVA (between subject effect: status [HYD/HYP]; within subject effect: time [PRE/POST]). Post hoc testing was conducted using Bonferroni correction. Additional covariates were included only if baseline testing indicated meaningful between-group differences, with parsimonious models favored. Partial eta squared (ηp^2^) with 95% CI was reported (approximate benchmarks: small ≈ 0.01, medium ≈ 0.06, large ≈ 0.14; Cohen, 1988), alongside adjusted means, standard error (SE), and 95% CI.

Mechanistic screening used Spearman rank correlations between changes and physiological markers. Time to 3% body-mass loss was analyzed with Kaplan-Meier estimators (event = 1 “reached 3%”, censored = 0) and groups compared by the log-rank test. Effect magnitude was quantified using a univariable Cox proportional-hazards model, reported as hazard ratios with 95% CI; proportional-hazards assumptions were assessed using Schoenfeld-type diagnostics and visual inspection of log-minus-log plots. Where medians were not reached within the follow-up, survival probabilities at prespecified times were reported. All analyses were performed in jamovi (version 2.6). Data visualizations were created in GraphPad Prism (version 9.4.5; GraphPad Software, LLC, San Diego, CA, USA). Statistical significance was set at two-sided α = 0.05.

## Results

### Baseline Characteristics

Groups were similar in stature, body mass, BMI, and the body surface area (BSA) (all *p* ≥ 0.73). Lean mass was moderately higher and the fat percentage moderately lower in the HYD group (g ≈ 0.50 and −0.61, respectively), but the differences did not reach significance. VO_2__max_ was higher in the HYD group by 5.10 ml·kg⁻^1^·min⁻^1^ (95% CI 1.23; 8.97; *p =* 0.01; *d* = 0.96). Descriptive results are presented in Table 1. At baseline (pre), there were no statistically significant between-group differences. Cognitive test results were all comparable between HYD and HYP groups (all *p* > 0.17).

**Table 1:** Baseline anthropometric, body-composition, and aerobic-capacity characteristics by hydration group.

Variable	HYD (n = 16)	HYP (n = 14)	Total (n = 30)
	M ± SD (±95%CI)		
Body height (cm)	180.0 ± 6.9 (176.3; 183.7)	179.3 ± 6.1 (175.7; 182.8)	179.7 ± 6.5 (177.3; 182.1)
Age (y)	34.25 ± 6.70 (30.68; 37.82)	35.14 ± 5.60 (31.91; 38.38)	34.67±6.12 (32.38; 36.95)
Body mass (kg)	81.0 ± 8.8 (76.3; 85.7)	80.3 ± 9.3 (74.9; 85.7)	80.6 ± 8.9 (77.3; 84.0)
BMI (kg·m⁻^2^)	25.0 ± 2.1 (23.9; 26.1)	24.9 ± 2.1 (23.7; 26.1)	24.9 ± 2.0 (24.2; 25.7)
Body surface area (m^2^)	2.01 ± 0.13 (1.93; 2.08)	2.00 ± 0.14 (1.91; 2.08)	2.00 ± 0.14 (1.95; 2.05)
Lean body mass (kg)	65.6 ± 7.9 (61.4; 69.9)	62.1 ± 5.2 (59.0; 65.1)	64.0 ± 6.9 (61.4; 66.6)
Body fat (%)	19.7 ± 4.2 (17.5; 22.0)	22.8 ± 5.7 (19.5; 26.1)	21.2 ± 5.1 (19.3; 23.1)
VO_2__max_ (ml·kg⁻^1^·min⁻^1^)	51.2 ± 6.2 (47.9; 54.5)	46.1 ± 4.0 (43.8; 48.4)	48.8 ± 5.8 (46.6; 51.0)
USG	1.009 ± 0.008 (1.005; 1.013)	1.019 ± 0.005 (1.016; 1.022)	1.014 ± 0.008 (1.011; 1.017)

M: mean; SD: standard deviation; CI: confidence interval; BMI: body mass index, calculated from body mass and stature (kg·m⁻^2^); VO_2__max_: maximal oxygen capacity

### Exercise Protocol Completion

**Figure 1:**
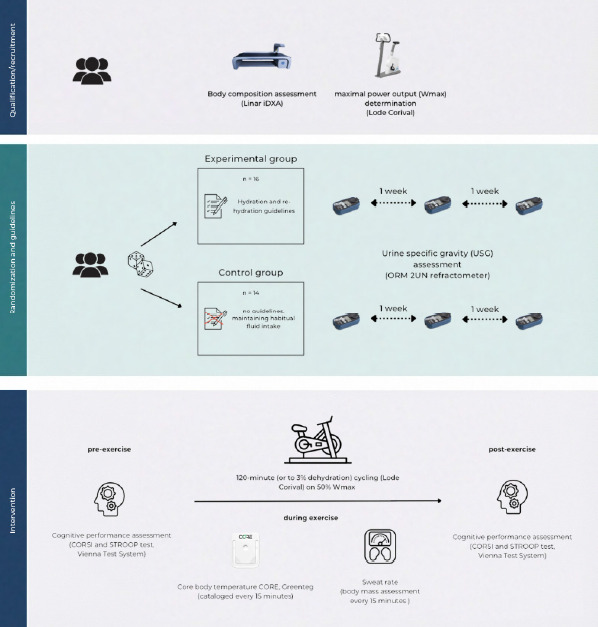
Study flowchart.

Kaplan-Meier curves for time to reach 3% body-mass loss (Figure 1) differed between groups (χ^2^ = 9.68, *p =* 0.002). In a univariable Cox model with hydration status as the predictor, HYP participants had a substantially lower risk of reaching the 3% threshold than the HYD group (HR = 0.08, 95% CI 0.01–0.61, *p =* 0.015), i.e., an ~92% reduction in instantaneous risk. Expressed inversely, the risk for the HYD group relative to the HYP group was ≈ 12.5-fold higher (95% CI ≈ 1.64–100). Consistently with this, 10/16 HYDRATED vs. 1/14 HYPOHYDRATED reached 3% within 120 min; the median time was not reached in the HYP group, whereas in the HYD group, it was ~120 min.

### Core Temperature

**Figure 2:**
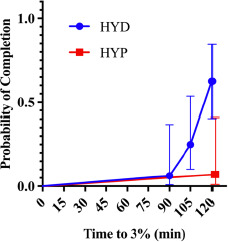
Kaplan-Meier curves for time to reach 3% body-mass loss during constant-load cycling by baseline hydration status (HYD vs. HYP). HYD: more optimally hydrated group, HYP: less optimally hydrated group

There was no evidence of a between-group difference between HYD and HYP groups for mean temperature 0–90 (°C·min) (U = 109.0, *p =* 0.918) (Figure 2A, Table 1).

Repeated-measures analysis of temperature across seven time points (pre, 15–90 min) in the HYD group showed a significant effect of time (χ^2^(6) = 73.3, *p* < 0.001, W = 0.76; Figure 2B, Table 1). Post hoc tests indicated higher temperature at every post-baseline point versus pre (all *p* < 0.001), with further increases from 15 to 30 (*p* = 0.036), 15 to 45/60/75/90 (all <0.001), 30 to 60/75/90 (all <0.001), 45 to 60/75/90 (all < 0.001), and 60 to 90 (*p =* 0.005) as well as 75 to 90 (*p =* 0.036); 30 to 45 and 60 to 75 were not significant (*p* = 0.168, 0.463).

Similar analysis in the HYP group showed a significant time effect (χ^2^(6) = 42.4, *p* < 0.001, W = 0.50; Figure 2B, Table 1). Temperature did not differ between pre vs. 15′ (*p* = 0.137), but was higher at 30′, 45′, 60′, 75′, 90′ vs. pre (*p* ≤ 0.003). Additional increases were observed for 15 to 45/60/75/90 (all *p* ≤ 0.008), 30 to 60/75/90 (*p* ≤ 0.016/0.001/ < 0.001), and 45 to 75/90 (*p =* 0.012/0.022), whereas 30 to 45, 45 to 60, 60 to 75, 60 to 90, 75 to 90 did not show significant differences (*p =* 0.283, 0.171, 0.233, 0.339, 0.811), indicating a rise to ~60–75′ followed by a relative plateau.

### Sweat Loss

**Figure 3:**
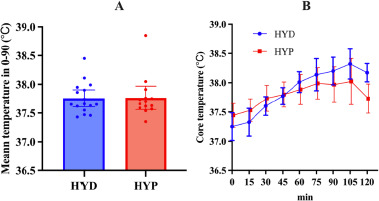
. Core temperature responses during constant-load cycling in HYD and HYP participants. HYD: more optimally hydrated group, HYP: less optimally hydrated group; A: mean core temperature over 0–90 min; B: core-temperature trajectories at 15-min intervals

The HYD group exhibited higher sweat production than the HYP group (Figure 3). For SR_mean_, the between-group difference was +0.304 l·h⁻^1^ (SE = 0.0606; 95% CI 0.18; 0.43), t(28) = 5.01, *p* < 0.001, *d* = 1.83 (95% CI 0.96; 2.68). For SR_peak_, the difference was +0.336 l·h⁻^1^ (SE = 0.1427; 95% CI 0.04; 0.63), t(28) = 2.35, *p =* 0.026, *d* = 0.86 (95% CI 0.10; 1.61).

### Cognitive Function

Analysis of the cognitive function outcome was conducted, controlling for the VO_2__max_ variable due to heterogeneity at baseline. Descriptive statistics for cognitive testing are shown in Tables 4 and 5.

**Table 2:** Core temperature at rest and during constant-load cycling by hydration group.

Variable	HYD (n = 16)	HYP (n = 14)
	Me ± [IQR] (min–max)	
Temp pre (°C)	37.31 [37.12–37.64] (36.03–37.97)	37.35 [37.29–37.52] (36.80–38.28)
Tc 15 min (°C)	37.37 [37.17–37.80] (36.09–38.08)	37.44 [37.37–37.60] (37.10–38.37)
Tc 30 min (°C)	37.52 [37.41–37.99] (37.29–38.64)	37.58 [37.52–37.80] (37.19–38.38)
Tc 45 min (°C)	37.66 [37.56–37.95] (37.43–38.36)	37.67 [37.62–37.84] (37.33–38.90)
Tc 60 min (°C)	38.01 [37.88–38.08] (37.58–38.99)	37.84 [37.67–37.89] (37.41–39.30)
Tc 75 min (°C)	38.05 [37.80–38.41] (37.31–39.40)	37.84 [37.73–37.92] (37.64–39.40)
Tc 90 min (°C)	38.16 [37.89–38.36] (37.76–39.54)	37.84 [37.78–37.98] (37.42–39.63)
Tc 105 min (°C)	38.33 [38.07–38.50] (37.58–39.55)	37.91 [37.75–38.00] (37.37–39.85)
Tc 120 min (°C)	38.16 [38.11–38.35] (37.71–38.52)	37.67 [37.63–37.83] (37.63–37.96)
Main effect of time	χ^2^(6) = 73.3, *p* < 0.001; W = 0.76	χ^2^(6) = 42.4, *p* < 0.001, W = 0.50
Mean Tc (°C)	37.66 [37.59–37.92] (37.43–38.45)	37.68 [37.60–37.76] (37.35–38.85)

Tc = core temperature (°C) at each time point; Mean Tc = within-participant average across available points. Values are presented as Me [IQR] (min–max) due to predominantly non-normal distributions

**Table 3:** Interval sweat rate and summary sweat-loss indices during constant-load cycling by hydration group.

Variable	HYD	HYP
	Me [IQR] (min–max)	
SR 0–15 (l·h⁻^1^)	0.80 [0.40–1.20] (0.40–1.60)	0.40 [0.40–0.80] (0.00–1.20)
SR 15–30 (l·h⁻^1^)	1.20 [0.80–1.20] (0.80–1.20)	0.80 [0.60–0.80] (0.40–1.20)
SR 30–45 (l·h⁻^1^)	1.20 [1.00–1.60] (0.80–1.60)	0.80 [0.80–1.20] (0.40–1.60)
SR 45–60 (l·h⁻^1^)	1.20 [1.20–1.60] (0.80–2.00)	0.80 [0.80–1.00] (0.80–1.20)
SR 60–75 (l·h⁻^1^)	1.20 [0.80–1.40] (0.80–2.00)	1.00 [0.80–1.20] (0.80–2.00)
SR 75–90 (l·h⁻^1^)	1.20 [1.20–1.40] (0.80–1.60)	1.10 [0.80–1.20] (0.80–1.60)
SR 90–105 (l·h⁻^1^)	1.20 [1.00–1.60] (0.80–2.40)	0.80 [0.60–1.00] (0.00–1.60)
SR_mean_ (l·h⁻^1^)	1.23 [1.09–1.29] (0.74–1.43)	0.89 [0.80–0.94] (0.63–1.09)
SR_peak_ (l·h⁻^1^)	1.60 [1.40–2.00] (0.80–2.40)	1.20 [1.20–1.60] (0.80–2.00)

SR denotes sweat rate (l·h⁻^1^) in consecutive 15-min windows (0–15, 15–30, …, 90–105); SR_mean_ = within-participant average across available windows; SR_peak_ = highest window value per participant. Values are reported as Me [IQR] (min–max) because most variables violated normality; hence, medians and quartiles were used instead of means and SD

**Table 4:** Stroop interference task outcomes (reaction times, inverse efficiency scores, and errors) before and after exercise by hydration group.

Variable	HYD		HYP	
	PRE	POST	PRE	POST
	M ± SD (±95%CI)			
Reaction time Interference-Reading (s)	0.10 ± 0.08 (0.06; 0.14)	0.08 ± 0,05 (0.05; 0,11)	0.08 ± 0.06 (0.04; 0.11)	0.04 ± 0,08 (−0.01–0.08)
Reaction time Interference-Naming (s)	0.07 ± 0.03 (0.05; 0.09)	0.08 ± 0,07 (0.04; 0.12)	0.05 ± 0.05 (0.02; 0.08)	0.0 ± 0.10 (0.05–0.16)
IES Interference-Reading	0.11 ± 0.08 (0.07–0.15)	0.00 ± 0.04 (−0.03–0.02)	0.08 ± 0.06 (0.05–0.12)	−0.01 ± 0.03 (−0.03–0.00)
IES Interference-Naming	0.07 ± 0.04 (0.05–0.09)	0.09 ± 0.09 (0.04–0.14)	0.06 ± 0.06 (0.02–0.09)	0.12 ± 0.10 (0.05–0.18)
Error interference-Reading (%)	6.67 ± 6.55 (3.17–10.16)	6.67 ± 5.96 (3.49–9.84)	6.67 ± 4.89 (3.84–9.49)	9.52 ± 7.38 (5.26–13.78)
Error interference-Naming (%)	5.63 ± 5.40 (2.74–8.51)	10.00 ± 7.79 (5.85–14.15)	7.38 ± 8.59 (2.42–12.34)	9.52 ± 8.56 (4.58–14.47)
	Me ± [IQR] (min–max)			
	PRE	POST	PRE	POST
Median RT-Reading (s)	0.95 [0.86–1.15] (0.78–1.52)	0.83 [0.78–1.01] (0.67–1.39)	0.95 [0.87–1.13] (0.72–1.32)	0.85 [0.79–0.98] (0.66–1.30)
Median RT-Naming (s)	0.81 [0.71–0.86] (0.68–1.27)	0.76 [0.71–0.85] (0.64–1.43)	0.79 [0.77–0.82] (0.64–0.99)	0.76 [0.71–0.88] (0.58–1.04)
Total errors-Reading	1.50 [1.00–2.00] (0.00–8.00)	2.00 [1.00–3.00] (0.00–7.00)	2.00 [1.00–3.00] (0.00–5.00)	2.50 [1.00–4.75] (0.00–7.00)
Total errors-Naming	1.00 [1.00–2.00] (0.00–5.00)	3.00 [1.00–4.25] (0.00–8.00)	1.00 [1.00–2.00] (0.00–8.00)	2.00 [1.00–3.75] (0.00–8.00)

RT: reaction time (s); IES: inverse efficiency score; CI: confidence interval; M: mean; SD: Standard deviation; IQR: interquartile range; min–max: range; ∆: change (POST-PRE)

**Table 5:** Corsi Block-Tapping Test performance (visuospatial working-memory indices) before and after exercise by hydration group.

Variable	HYD		HYP	
	PRE	POST	PRE	POST
	M ± SD (±95%CI)			
BT (ms)	404.75 ± 135.37 (332.62; 476.88)	402.75 ± 121.38 (338.07; 467.43)	404.07 ± 108.35 (341.51; 466.63)	447.50 ± 106.12 (386.23; 508.77)
RSS	5.06 ± 0.68 (4.70; 5.42)	5.31 ± 0.60 (4.99; 5.63)	5.29 ± 0.8 (4.81; 5.76)	5.64 ± 0.93 (5.11; 6.18)
SWM	5.56 ± 0.81 (5.13; 6.00)	5.88 ± 1.20 (5.23; 6.52)	6.07 ± 1.14 (5.41; 6.73)	6.14 ± 0.77 (5.70; 6.59)
Best span	6.19 ± 1.17 (5.57; 6.81)	6.44 ± 1.03 (5.89; 6.99)	6.36 ± 1.15 (5.69; 7.02)	6.93 ± 1.00 (6.35; 7.50)
Raw score	10.25 ± 2.18 (9.09; 11.41)	11.06 ± 2.49 (9.74; 12.39)	11.50 ± 2.77 (9.90; 13.10)	12.36 ± 2.27 (11.04; 13.67)
	Me ± [IQR] (min–max)			
Incorrect trials	4.50 [3.75–5.00] (3.00–7.00)	4.00 [3.00–6.00] (3.00–7.00)	4.00 [3.00–4.00] (3.00–7.00)	4.00 [3.00–5.00] (3.00–9.00)
Order errors	2.50 [2.00–3.00] (0.00–4.00)	2.00 [1.75–3.00] (0.00–5.00)	2.50 [2.00–3.00] (1.00–5.00)	3.00 [2.00–3.00] (1.00–5.00)

BT: Block time; RSS: Scaled score; SWM: Spatial working memory; SD: Standard deviation; CI: Confidence interval; Me: Median; IQR: Interquartile range; min–max: range

### Error Interference

A repeated-measures ANCOVA on error interference in reading showed no significant main effect of time, F(1,27) = 0.284, *p =* 0.599, no time × status interaction, F(1,27) = 0.894, *p =* 0.353.

A repeated-measures ANCOVA on error interference in naming yielded the following results: time, F(1,27) = 0.000, *p =* 0.988; time × status, F(1,27) = 0.246, *p =* 0.624.

### Reaction Time Interference

A repeated-measures ANCOVA on reading reaction time interference showed the following results: time, F(1,27) = 0.124, *p =* 0.728; time × status, F(1,27) = 0.210, *p =* 0.650.

On the other hand, a repeated-measures ANCOVA on naming reaction time interference showed no significant main effect of time, F(1,27) = 2.000, *p =* 0.169, but a significant time × status interaction, F(1,27) = 4.570, *p =* 0.042, η^2^_p_ = 0.145, and a non-significant trend for the time × VO_2__max_ interaction, F(1,27) = 2.770, *p =* 0.107 (Figure 5A). Bonferroni-corrected post hoc comparisons indicated that the HYD and HYP groups did not differ at baseline (*p =* 1.000), and the pre-to-post change in naming interference within the HYD group was trivial (*p =* 1.000). In contrast, the HYP group showed a clear increase in naming interference from pre to post (Δ = 0.064, *p =* 0.029, *d* = 0.80).

**Figure 4:**
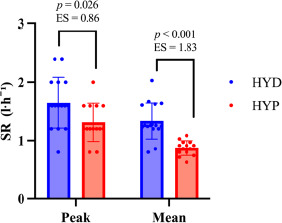
. Between-group differences in the mean and peak sweat rate during constant-load cycling in HYD and HYP participants. HYD: more optimally hydrated group; HYP: less optimally hydrated group, SR: sweat rate, Peak: Peak sweat rate; Mean: Mean Sweat rate; ES: effect size, p: p-value

**Figure 5:**
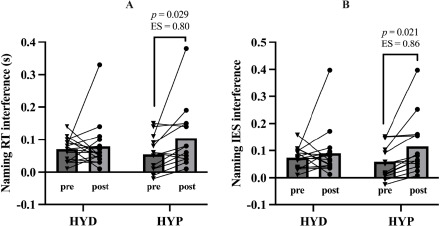
Changes in naming interference (A: reaction time and B: inverse efficiency score) before and after exercise in HYD and HYP participants. HYD: more optimally hydrated group; HYP: less optimally hydrated group; RT: reaction time; ES: effect size, p: p-value

### IES Interference

A repeated-measures ANCOVA on the naming IES showed no main effect of time, F(1,27) = 2.620, *p =* 0.117, a significant time × status interaction, F(1,27) = 4.510, *p =* 0.043, η^2^_p_ = 0.143, no time × VO_2__max_ interaction, F(1,27) = 3.600, *p =* 0.069, η^2^_p_ = 0.118 (Figure 5B). Bonferroni-corrected post hoc comparisons indicated that the HYD and HYP groups did not differ at baseline (*p =* 1.000), and the pre-post change in the HYD group was trivial and non-significant (*p =* 1.000). In contrast, the HYP group showed a marked pre-post change in the IES (Δ = 0.071, *p =* 0.021, *d* = 0.86). The comparison between groups at post yielded a non-significant difference (*p =* 0.581).

IES reading showed a significant main effect of time, F(1,27) = 5.790, *p =* 0.023, η^2^_p_ = 0.177, with no time × status interaction, F(1,27) = 1.590, *p =* 0.218, η^2^_p_ = 0.056, and no time × VO_2__max_ interaction, F(1,27) = 2.950, *p =* 0.097, η^2^_p_ = 0.099. Between-subjects effects were not significant for hydration status, F(1,27) = 2.118, *p =* 0.157, η^2^_p_ = 0.073, nor for VO_2__max_, F(1,27) = 0.145, *p =* 0.706, η^2^_p_ = 0.005. IES reduced overall (∆ = 0.105, *p =* 0.001, *d* = 2.21).

For block time, there was no significant main effect of time, F(1,27) = 3.230, *p =* 0.083, and no significant time × status interaction, F(1,27) = 3.020, *p =* 0.094. For the scaled score, no main effect of time was observed, F(1,27) = 1.396, *p =* 0.248, and no time × status interaction, F(1,27) = 0.008, *p =* 0.931. For spatial working memory, there was no main effect of time, F(1,27) = 0.044, *p =* 0.836, and no time × status interaction, F(1,27) = 0.438, *p =* 0.514. For the best span, there was a trend-level main effect of time, F(1,27) = 4.130, *p =* 0.052, with no significant time × status interaction, F(1,27) = 3.260, *p =* 0.082, and a significant time × VO_2__max_ interaction, F(1,27) = 5.190, *p =* 0.031. For the raw score, no significant main effect of time, F(1,27) = 0.542, *p =* 0.468, and no time × status interaction, F(1,27) = 0.273, *p =* 0.606, were detected.

### Associations

All correlations between cognitive outcomes (and changes) and mechanistic markers were non-significant (all *p* > 0.05). The same held when analyses were run separately within each group (HYD and HYP): no correlation reached significance (all *p* > 0.05).

## Discussion

This study evaluated whether baseline hydration status would modulate thermoregulatory and cognitive responses during prolonged cycling under thermoneutral conditions. Consistently with prior research and commonly accepted views that hypo hydration reduces endurance tolerance and can blunt heat-dissipating responses as dehydration progresses (Cheuvront and Kennefick, 2014; Périard et al., 2021; Sawka et al., 2007), the main finding was that participants who began exercise in a more hydrated state (HYD) exhibited elevated sweat rates throughout the exercise bout (ΔSR_mean_ ≈ +0.30 L·h⁻^1^; Δ SR_peak_ ≈ +0.34 L·h⁻^1^) and tended to accumulate a greater core temperature over time. In contrast, early Tc burden did not differ clearly between groups, which aligns with evidence that absolute metabolic heat production and aerobic fitness can dominate Tc and sudomotor responses when intensity is prescribed relatively (e.g., %W_max_), underscoring the importance of matching heat production in between-group comparisons (Sawka et al., 2001; Jay and Cramer, 2015). Finally, the selective worsening of Stroop interference with minimal change in Corsi outcomes is consistent with reports that dehydration more consistently impacts executive control than visuospatial working memory (Deshayes et al., 2022; Watanabe et al., 2024; Wittbrodt and Millard-Stafford, 2018).

### Hydration Status and Thermoregulation

Changes in the rate of Tc rise did not support the hypothesis that individuals with insufficient hydration status would exhibit a greater increase in core temperature. Two features likely explain the paradox of higher sweating yet greater core-temperature rise in the HYD group. First, HYD participants had higher aerobic capacity (VO_2__max_ +5.1 mL·kg⁻^1^·min⁻^1^), which implies greater absolute metabolic heat production at the standardized relative workload (50% W_max_), because absolute work (W) scales with each individual’s W_max_. Greater heat production elicits stronger sudomotor and skin blood-flow drive, increasing both the sweat rate and the core-temperature trajectory, especially early before evaporative balance is fully established. This pattern aligns with comparative thermoregulation work showing that aerobic fitness and the absolute rate of metabolic heat production are primary determinants of sweating and core-temperature responses, and that fair comparisons across groups often require matching heat production rather than only relative intensity (Jay and Cramer, 2015). The overall direction is also consistent with classic models, indicating that, for a given thermal environment, higher internal heat production raises the defended core temperature unless fully offset by evaporation (Sawka et al., 2001).

Conversely, initial hypo hydration is known to attenuate the skin blood flow and sweating at a given core temperature via the combined effects of hypovolemia and hypertonicity, thereby diminishing evaporative capacity and exacerbating heat storage at matched heat production (Sawka et al., 2001). However, HYP participants executed a lower absolute workload, which likely limited metabolic heat production and, in part, accounted for their smaller rise in core temperature despite suboptimal hydration.

The markedly lower protocol completion rate (survival to 3% body-mass loss) indicates diminished exercise tolerance rather than enhanced thermal safety. The significantly lower probability that HYP participants reached the 3% body-mass loss threshold (and thus completed the full 120 min) indicates that hypo hydration increases cardiovascular and perceptual strain and diminishes time to exhaustion under continuous work, even in neutral temperate settings (Cheuvront et al., 2003; Pilch et al., 2022). In practical terms, individuals commencing exercise in a suboptimal hydration state are more likely to self-limit or terminate the bout prematurely, which plausibly accounts for decreased performance observed after 90 min in our dataset and justifies restricting primary thermoregulatory analyses to 0–90 min to mitigate informative dropout bias.

Our findings on hydration and exercise thermoregulation are consistent with the literature. Hypo hydration typically impairs heat dissipation (reduced sweating and skin blood flow for a given Tc) and reduces endurance performance and tolerance, especially once body-mass loss exceeds ~2% (Cheuvront and Kennefick, 2014; Périard et al., 2021). The present data extend this by emphasizing baseline hydration before exercise as starting in a more optimal hydration state was associated with a more robust sudomotor response, but, because of higher absolute work, also a larger core- temperature burden. That interaction between baseline hydration and exercise dehydration is under-reported in the literature. Methodologically, recent thermo-physiology work advocates matching the rate of metabolic heat production or normalizing to body size when comparing groups, and our data illustrate why such rigor is essential when baseline VO_2__max_ values differ (Jay and Cramer, 2015).

### Hydration Status and Neurocognitive Performance

The selective impairment on Stroop interference in HYP participants (≈+63 ms slower) is directionally consistent with meta-analytic evidence that dehydration affects more executive functions (attention/inhibition) than simple speeded responses or short-term visuospatial memory, with effects most apparent beyond ~2% body-mass loss or when dehydration is sustained (Wittbrodt and Millard-Stafford, 2018). A recent study on sustained mild dehydration likewise reports prolonged Stroop response times despite minimal changes in autonomic thermoregulation, suggesting central/cognitive vulnerability even when core temperature is not dramatically elevated (Watanabe et al., 2024). By contrast, we observed no between-group as well as no pre-post differences in the Corsi block-tapping task, which is consistent with systematic evidence that visuospatial working-memory performance is often unchanged immediately after exercise-induced hypo hydration of ~2–4% (Deshayes et al., 2022).

The lack of robust correlations between thermoregulatory metrics and cognitive decrements suggests that circulating volume/osmolality and cerebral perfusion/neurotransmission (rather than core temperature per se) could be proximal drivers of the executive-function effect, an interpretation aligned with mechanistic data (Zhang et al., 2019). It therefore appears that commencing exercise in a suboptimal hydration state confers a substantially greater risk of executive dysfunction, particularly with respect to inhibitory control, than of impairments in working memory.

### Methodological Considerations

Continuous core temperature was recorded using a non-invasive heat-flux-based device. While this approach enables fine-grained temporal profiling, published validations are mixed: some reports question agreement with rectal and intra gastrointestinal references beyond ~0.3°C under dynamic heat loads, whereas others indicate their utility yet with careful protocols (Verdel et al., 2021). Our conclusions focused on between-group patterns and within-subject trajectories rather than absolute Tc, mitigating this concern. Nonetheless, future work should triangulate with ingestible or esophageal measures where feasible.

The group difference in VO_2__max_ is both an ecological strength (reflecting real-world heterogeneity) and an analytical challenge. Since the absolute workload was not equated, aerobic fitness likely mediated part of the observed association between hydration and thermoregulation. Future studies could strengthen causal inference by matching metabolic heat production across groups or incorporating heat-production estimates as covariates.

### Practical Implications

A key practical implication of our findings is the strong need to assess and manage hydration status prior to exercise. Routine checks, such as urine specific gravity along with urine color and, where greater rigor is required, plasma/serum and urine osmolality or blood copeptin concentration can help estimate hydration status and thereby reduce the risk of diminished exercise tolerance and decrements in cognitive performance.

### Limitations

This study has several limitations. First, participants were male and tested under thermoneutral laboratory conditions, limiting external validity to women and contexts involving heat stress. Second, core temperature was estimated with the CORE device. Although previously validated, this method may introduce device-specific bias relative to ingestible or esophageal thermometry (Januário et al., 2024; Mündel et al., 2016). Third, hydration status was estimated by urine specific gravity, as plasma osmolality was not measured, limiting granularity regarding acute versus chronic hydration. Fourth, the sweat rate was derived from mass balance rather than direct sweat collection, potentially reducing estimation precision. Fifth, despite familiarization, residual practice/learning effects on cognitive outcomes cannot be fully excluded. Finally, between-group differences in VO_2__max_ may have influenced metabolic heat production.

### Future Directions

Equating metabolic heat production across hydration status, integrating direct plasma volume/osmolality metrics, and including cerebral blood flow or neurovascular measures would clarify the pathways linking baseline hydration to both heat strain and executive function decrements. Expanding to hotter, more humid, and clothing-encumbered contexts will improve ecological validity for field sports and occupational settings.

## Conclusions

Baseline hydration status influenced both thermoregulation and exercise tolerance. Better-hydrated participants showed higher sweat rates and were far more likely to reach 3% body-mass loss, yet mean core-temperature burden over 0–90 min did not differ between groups. A trend toward greater core-temperature rise in the euhydrated group may reflect higher absolute metabolic heat production in fitter HYD participants exercising at the same relative intensity. Hydration status selectively affected cognition: insufficiently hydrated individuals demonstrated slower Stroop naming interference, whereas visuospatial working memory (Corsi) was unchanged. Across the sample, changes in executive performance were not significantly related to thermoregulatory strain, implicating fluid-balance processes rather than core temperature per se as the more proximal driver of the cognitive effect.

Practically, these data support routine verification and optimization of pre-exercise hydration status to preserve exercise tolerance and executive function. Methodologically, future studies should control or model metabolic heat production as well as include direct fluid-balance and cerebrovascular measures to refine causal inference.
